# Trends and inequalities in exclusive breastfeeding among 0–5 month olds in Ghana from 1993 to 2022

**DOI:** 10.3389/fpubh.2025.1702411

**Published:** 2026-01-08

**Authors:** Patience Fakornam Doe, Amidu Alhassan, Gifty Serwaa Amponsah, Boahemaa Adu Otchere, Frank Offei Odonkor, Gloria Kossi Lartey, Abraham Norman Nortey, Joseph Lasong, Yula Salifu

**Affiliations:** 1 Department of Public Health, School of Nursing and Midwifery, College of Health and Allied Sciences, University of Cape Coast, Cape Coast, Ghana; 2 Department of Adult Health, School of Nursing and Midwifery, College of Health and Allied Sciences, University of Cape Coast, Cape Coast, Ghana; 3 Department of Community Medicine, School of Medical Sciences, University of Cape Coast, Cape Coast, Ghana; 4 Department of Population and Reproductive Health, School of Public Health, University for Development Studies, Tamale, Ghana; 5 Department of Population, Family and Reproductive Health, School of Public Health, University of Ghana, Accra, Ghana

**Keywords:** exclusive breastfeeding, Ghana, inequalities, infant nutrition, maternal health

## Abstract

**Background:**

Exclusive breastfeeding (EBF) during the first 6 months of life is essential for reducing infant morbidity and mortality, enhancing immunity, and supporting maternal health. Despite global and national commitments, progress remains uneven. This study examined long-term trends and inequalities in EBF among infants aged 0–5 months in Ghana between 1993 and 2022.

**Methods:**

A secondary analysis was conducted using nationally representative data from nine rounds of the Ghana Demographic and Health Surveys (GDHS) and Multiple Indicator Cluster Surveys (MICS). The outcome was EBF prevalence, defined according to WHO and UNICEF guidelines. Inequalities were examined by maternal age, education, household wealth, residence, child sex, and region, using both simple (absolute difference, ratio) and complex measures (population attributable fraction, population attributable risk, and absolute concentration index).

**Results:**

National EBF prevalence rose from 5.8% in 1993 to 53.1% in 2022, peaking at 62.8% in 2008, before stabilising. Changes in inequality patterns were observed. Earlier positive gradients by wealth and education evolved into U-shaped distributions, with the lowest and highest groups reporting higher prevalence than the middle categories. Despite these shifts, cross-sectional inequalities remained modest in magnitude relative to the considerable national increase. Wealth- and residence-related gaps narrowed over time, although regional disparities remained pronounced.

**Conclusion:**

Ghana achieved major improvements in exclusive breastfeeding over three decades, while socio-demographic inequalities were comparatively small and did not account for the recent plateau. The emergence of U-shaped patterns in wealth and education underscores the need for tailored interventions that reach middle socioeconomic groups and regions with persistently low prevalence. Strengthening equity-focused and context-specific strategies is essential for sustaining progress towards Sustainable Development Goal 3.

## Introduction

Exclusive breastfeeding (EBF) for the first 6 months of life is globally recognised as a cornerstone of infant and maternal health. Globally, only about 48% of infants under 6 months were exclusively breastfed in 2022 (1). In sub-Saharan Africa, the average prevalence is similar at about 49%, but this masks substantial variation, with rates as high as 83% in Burundi and as low as 19% in Gabon (2). These figures underscore persistent disparities in infant feeding practices, with serious implications for survival, growth, and long-term development.

In Ghana, trends in EBF reflect both progress and regression. The rate was as low as 7.4% in 1993 but peaked at 63% in 2008 (3), reflecting improvements in breastfeeding promotion. However, the Ghana Multiple Indicator Cluster Survey (MICS) showed a decline to 43% in 2017–2018 (4), and the 2022 Ghana Demographic and Health Survey (GDHS) reported that only 53% of infants under 6 months were exclusively breastfed, representing almost no improvement from the 52% recorded in 2014 (5). A meta-analysis further estimated a pooled national prevalence of 50% (3). Despite intermittent gains, Ghana remains off track in achieving national and global breastfeeding targets.

EBF provides infants with the ideal balance of nutrients and bioactive factors essential for growth and development, while strengthening immunity against infections such as diarrhoea and pneumonia, two leading causes of under-five mortality (6, 7). Beyond infancy, EBF has been linked to improved cognitive outcomes, reduced risk of obesity, and protection against chronic diseases later in life (8, 9). For mothers, it offers protection against postpartum haemorrhage, accelerates recovery, and reduces the lifetime risk of breast and ovarian cancers (8, 9). Collectively, these benefits demonstrate that EBF is one of the most cost-effective interventions for improving child survival and maternal well-being, making its promotion a critical priority in global health agendas (10).

The persistence of relatively low EBF rates, particularly among Ghanaian women, raises critical public health concerns. Exclusive breastfeeding has proven benefits in reducing infant morbidity, strengthening immunity, and improving maternal health outcomes (11). However, contextual barriers hinder its practice such as traditional beliefs (12, 13), early introduction of water in hot climates, remains widespread (14, 15). Structural barriers, such as limited maternity leave, usually 3 months, undermine women’s ability to sustain EBF for the recommended 6 months (16). These socio-cultural and economic dynamics exacerbate inequalities, disproportionately affecting mothers in urban and low-income settings, thereby undermining child health equity.

Despite strong evidence of the health benefits of exclusive breastfeeding and its prioritisation within SDG 3.2, Ghana’s progress has stagnated. National surveys show little improvement over the past decade, with EBF prevalence standing at only 53% in 2022 (5). While research highlights factors such as cultural practices (12, 13), inadequate maternity protection, and insufficient institutional support (17), there is limited evidence on how these determinants interact across socio-demographic groups and regions. This gap weakens policy formulation and programme design, leaving vulnerable populations without tailored interventions. In turn, such deficiencies risk perpetuating preventable infant morbidity and mortality, slowing progress towards the SDG 3.2 target. This study aims to examine trends and inequalities in exclusive breastfeeding among infants aged 0–5 months in Ghana from 1993 to 2022. Specifically, it explored disparities by residence, and socio-demographic factors, while exploring contextual influences that shape feeding practices. The evidence generated will be vital for policymakers, programme implementers, and healthcare providers, enabling targeted strategies such as enhanced maternity protection, culturally sensitive breastfeeding counselling, and supportive workplace policies. This study seeks to address key evidence gaps, thereby contributing to Ghana’s progress towards the realisation of SDG 3.2: the elimination of preventable mortality among neonates and children under 5 years, facilitated through advancements in nutrition and health system strengthening.

## Methods

### Study design

This study adopted a secondary analysis of nationally representative cross-sectional surveys conducted in Ghana between 1993 and 2022. The focus was on EBF practices among infants aged 0–5 months. The use of repeated cross-sectional datasets enabled the examination of temporal trends and inequalities across sociodemographic and regional subgroups, ensuring comparability over three decades. All indicators were obtained exclusively through the WHO HEAT platform. The dataset used, therefore, comprised pre-processed indicators generated by WHO HEAT based on microdata originally collected by the DHS and MICS programmes. No raw DHS or MICS household or child-level data were accessed for this analysis. This ensures comparability across survey waves, although it limits the researcher’s control over indicator construction.

### Study setting

The study was conducted in Ghana, a lower-middle-income country in West Africa with 16 administrative regions. These are Ahafo, Ashanti, Bono, Bono East, Central, Eastern, Greater Accra, Northern, Northeast, Oti, Savannah, Upper East, Upper West, Volta, Western, and Western North (18). The regions differ in population distribution, economic conditions, health-service availability, and infrastructure. Poverty levels remain highest in the northern ecological zones, while the southern urban corridor records better access to services and improved socioeconomic indicators (18). The health system follows a tiered structure comprising community-based services, primary care facilities, district hospitals, and regional referral centres (19). The Community-based Health Planning and Services programme strengthens primary care delivery and supports maternal and child health interventions, particularly in rural settings (18). Breastfeeding promotion is guided by national policies such as the Infant and Young Child Feeding Strategy and the Baby-Friendly Hospital Initiative (20).

### Data sources

Data were obtained from the Ghana Demographic and Health Surveys (GDHS) and the Ghana Multiple Indicator Cluster Surveys (MICS). The GDHS are implemented by the Ghana Statistical Service (GSS) and the Ghana Health Service (GHS) with technical support from the Demographic and Health Surveys (DHS) Programme, while MICS are coordinated by GSS in collaboration with UNICEF through the WHO Health Equity Toolkit platform. Both survey programmes employ standardised sampling procedures, interviewer training, and data collection instruments to ensure cross-country comparability and reliability. For this analysis, nine survey waves were included: 1993, 1998, 2003, 2006, 2008, 2011, 2014, 2017, and 2022.

### Study population

The analytic population comprised infants aged 0–5 months and their mothers interviewed in the DHS and MICS rounds. Inclusion was based on the availability of breastfeeding information recorded during the survey reference period. Key maternal and household characteristics were extracted, including maternal age (15–19 and 20–49 years), maternal education (no education, primary, secondary, and higher), household wealth quintile (derived through principal component analysis of assets), place of residence (urban/rural), child sex, and administrative region.

### Outcome variable

The primary outcome was exclusive breastfeeding prevalence, defined in line with World Health Organization (WHO) and UNICEF recommendations. Infants 0–5 months were considered exclusively breastfed if they received only breast milk in the 24 h preceding the survey, with exceptions for oral rehydration solution, medicines, and vitamin/mineral supplements (21).

### Measures of inequality

Inequalities in EBF were assessed using both simple and complex summary measures, following the WHO Health Inequality Monitoring framework (WHO, 2022). Simple measures included the absolute difference (D) and ratio (R) between advantaged and disadvantaged groups. Complex measures included the population attributable fraction (PAF), population attributable risk (PAR), and the absolute concentration index (ACI), which account for the distribution of EBF across the entire population. These metrics were calculated across wealth quintiles, maternal education, age, sex of the child, place of residence, and region.

### Data analysis

Data analysis was performed using STATA version 17/MP (StataCorp, College Station, TX, United States). National prevalence estimates were calculated for each survey year, with disaggregation by sociodemographic variables. Trends were assessed through descriptive statistics and graphical displays. Inequality measures were computed using established WHO and UNICEF methodologies. All analyses applied sampling weights provided by DHS and MICS to correct for unequal probabilities of selection, and Taylor series linearization was used to adjust for complex survey design effects. Prevalence estimates were presented with 95% confidence intervals (CI) to account for sampling variability and ensure statistical reliability.

## Results

### National prevalence of exclusive breastfeeding among 0–5 month olds

**Figure 1:**
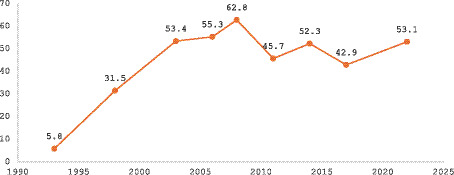
National prevalence of exclusive breastfeeding among 0–5 month olds.

Figure 1 shows a marked rise in EBF among infants aged 0–5 months in Ghana. Prevalence increased steeply from 5.8% in 1993 to 62.8% in 2008, representing the largest historical gain, before stabilising with minor fluctuations through to 53.1% in 2022. This pattern demonstrates substantial early progress followed by a plateau in the past decade.

### Trends and inequalities in exclusive breastfeeding among 0–5 month olds in Ghana by education, place of residence, and sex

**Figure 2:**
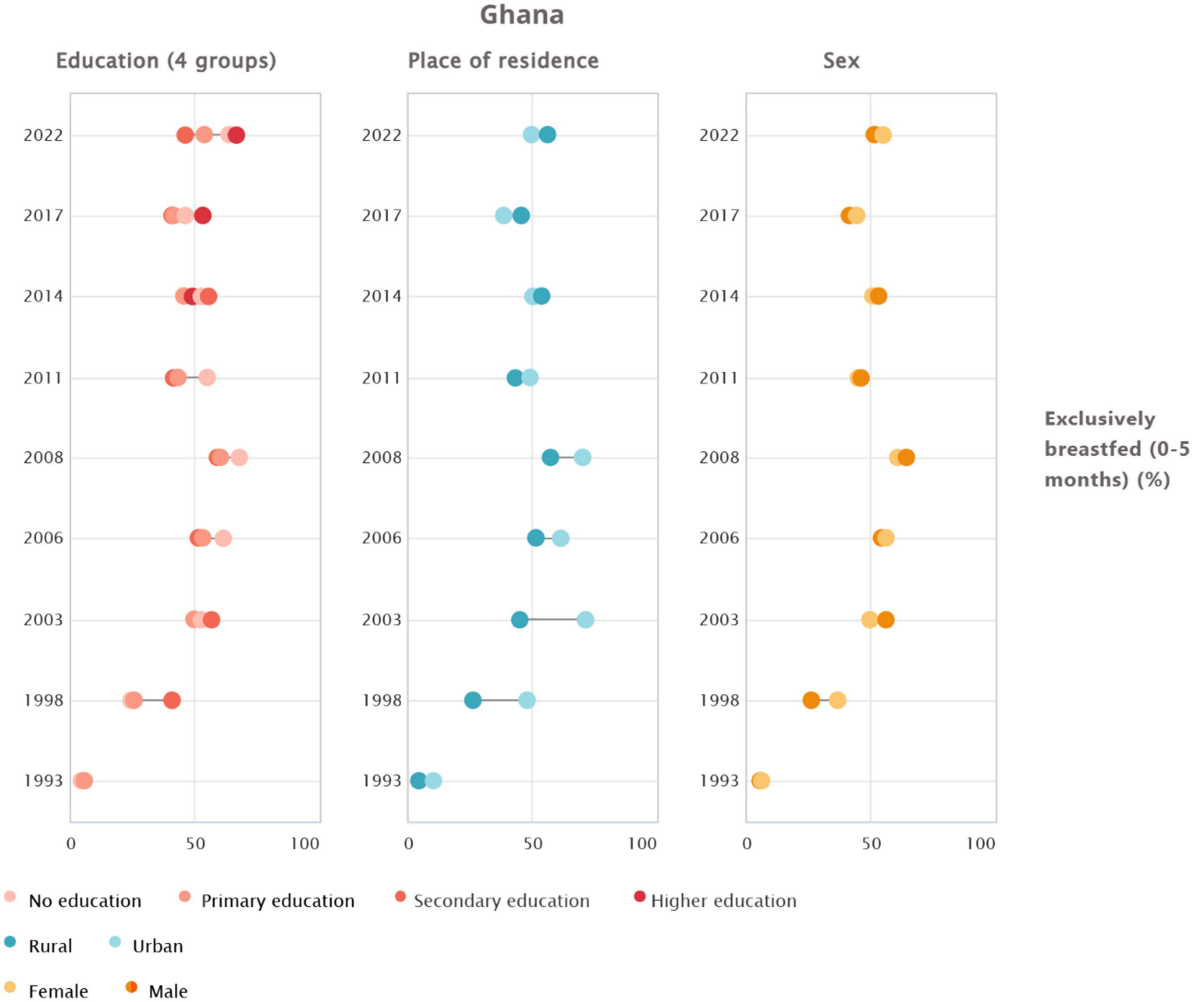
Trends and inequalities in exclusive breastfeeding among 0–5 month olds in Ghana by education, place of residence, and sex.

The data from 1993 to 2022 reveal marked disparities in EBF by education, residence, and sex. Educational differences no longer follow a simple gradient. Instead, a U-shaped pattern has emerged, with mothers without schooling and those with higher education showing comparatively high EBF, while the middle categories record lower rates. Residence-based differences have shifted over time. Urban mothers initially had a clear advantage, but this pattern reversed from 2014 onwards, with rural mothers now reporting higher prevalence; in 2022, EBF reached 55.97% in rural areas compared with 49.52% in urban settings. Sex differences are modest. Girls currently have slightly higher EBF than boys, although the historical pattern has not been consistent. Across all groups, levels have risen steadily, reflecting broad national progress despite changing inequality patterns (Figure 2).

### Trends and inequalities in exclusive breastfeeding among 0–5 month olds in Ghana across different age groups and economic status (wealth quintiles)

**Figure 3:**
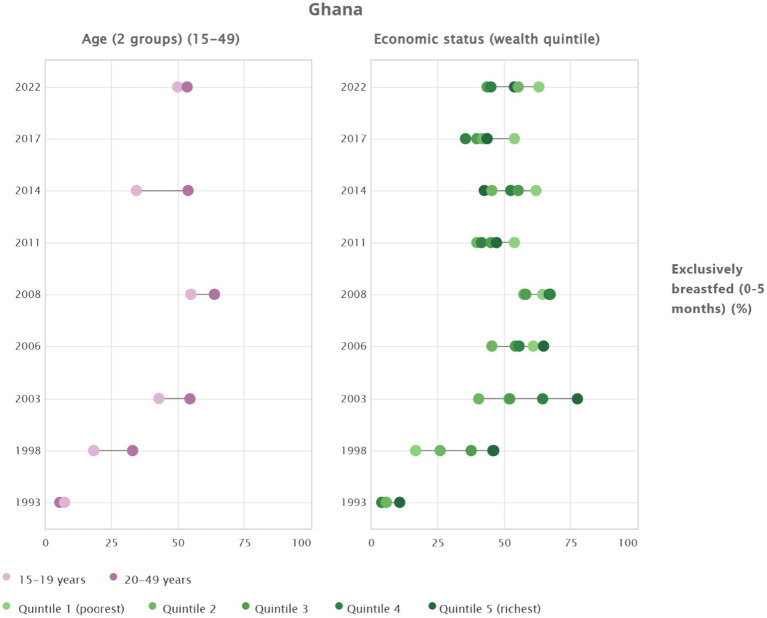
Trends and inequalities in exclusive breastfeeding among 0–5 month olds in Ghana across different age groups and economic status (wealth quintiles).

Exclusive breastfeeding hows marked differences by age and economic status in Ghana. Mothers aged 20–49 years consistently achieve higher EBF than those aged 15–19 years, confirming that adolescent mothers maintain lower uptake across survey rounds. Wealth patterns have shifted over time. Earlier linear gradients have transformed into a U-shaped relationship, with the poorest and richest groups reporting higher EBF, while the middle quintiles remain the lowest. In 2022, the poorest quintile recorded the highest prevalence, whereas Quintiles 2 and 3 showed the weakest performance. Long-term trends indicate improvement across all groups from 1993 to 2022, yet the persistent disadvantage among adolescents and middle wealth categories highlights unequal progress that requires targeted intervention (Figure 3).

### Trends and inequalities in exclusive breastfeeding among 0–5 month olds in Ghana from 1993 to 2022 across sociodemographic groups

Exclusive breastfeeding in Ghana has shown remarkable improvement between 1993 and 2022, yet inequalities persist across sociodemographic groups (Table 1). Among adolescents aged 15–19 years, prevalence rose substantially from 7.14% (95% CI: 2.27–20.28) in 1993 to 49.87% (95% CI: 35.84–63.91) in 2022, while among women aged 20–49 years, it increased from 5.64% (95% CI: 3.57–8.80) to 53.39% (95% CI: 48.89–57.84). Socioeconomic disparities were evident: women in the poorest quintile reported an increase from 3.90% (95% CI: 1.21–11.80) to 63.22% (95% CI: 56.14–69.78), eventually surpassing the richest quintile, which moved from 10.71% (95% CI: 4.79–22.23) to 54.05% (95% CI: 41.44–66.17). Education followed a similar trend, with women without formal schooling increasing from 4.40% (95% CI: 2.04–9.25) to 63.74% (95% CI: 56.08–70.76), while those with higher education achieved the highest prevalence of 67.01% (95% CI: 51.09–79.79) in 2022. Urban–rural dynamics also shifted: although urban mothers initially had an advantage, rising from 10.10% (95% CI: 5.28–18.45) to 49.52% (95% CI: 42.43–56.64), rural mothers eventually overtook them, improving from 4.29% (95% CI: 2.31–7.81) to 55.97% (95% CI: 50.64–61.16). Differences by sex of the child were modest, with females slightly more likely to be exclusively breastfed (55.18, 95% CI: 48.56–61.62) than males (51.47, 95% CI: 45.72–57.17) in 2022. However, regional variations were profound in 2022, ranging from as low as 27.5% (95% CI: 17.2–41.0) in Western North to as high as 78.1% (95% CI: 65.4–87.1) in Savannah (Table 1). Collectively, these findings highlight significant national progress in EBF over three decades, alongside enduring disparities shaped by geography, wealth, education, and age.

**Table 1:** Trends and inequalities in exclusive breastfeeding among 0–5 month olds in Ghana, from 1993–2022 across sociodemographic groups.

Dimension	1993		1998		2003		2006		2008		2011		2014		2017		2022	
	N	Est. (LB–UB)	N	Est. (LB–UB)	N	Est. (LB–UB)	N	Est. (LB–UB)	N	Est. (LB–UB)	N	Est. (LB–UB)	N	Est. (LB–UB)	N	Est. (LB–UB)	N	Est. (LB–UB)
Age																		
Age (15–19)	42	7.14 (2.27–20.28)	28	18.20 (7.45–38.07)	30	43.08 (27.55–60.11)	–	–	29	54.93 (35.53–72.94)	–	–	43	34.47 (20.13–52.32)	–	–	67	49.87 (35.84–63.91)
Age (20–49)	337	5.64 (3.57–8.80)	263	32.94 (27.34–39.08)	278	54.52 (48.04–60.85)	–	–	279	63.63 (57.66–69.21)	–	–	518	53.77 (48.46–58.99)	–	–	759	53.39 (48.89–57.84)
Quintile 1 (Poorest)	77	3.90 (1.21–11.80)	85	16.87 (10.07–26.89)	76	51.86 (40.35–63.18)	81	60.85 (46.39–73.62)	72	64.62 (52.30–75.27)	169	53.75 (44.94–62.34)	138	62.05 (52.49–70.75)	169	53.99 (45.70–62.08)	215	63.22 (56.14–69.78)
Quintile 2	101	5.94 (2.68–12.63)	64	26.05 (17.05–37.66)	80	40.32 (29.04–52.72)	100	45.57 (35.56–55.96)	63	57.39 (45.16–68.78)	163	39.72 (30.56–49.66)	110	45.29 (35.35–55.62)	166	41.98 (31.52–53.21)	172	55.33 (46.70–63.65)
Quintile 3	74	5.41 (2.15–12.91)	55	37.64 (24.20–53.29)	78	52.18 (39.72–64.37)	63	54.12 (39.39–68.17)	61	58.09 (44.29–70.73)	186	45.08 (33.66–57.04)	117	55.18 (43.33–66.46)	181	39.71 (29.55–50.84)	144	43.50 (32.28–55.44)
Quintile 4	71	4.23 (1.32–12.73)	50	45.82 (33.19–59.01)	40	64.56 (48.20–78.10)	81	55.79 (41.22–69.43)	69	67.46 (55.07–77.81)	135	41.66 (30.23–54.05)	102	52.30 (39.42–64.88)	171	35.46 (26.35–45.77)	164	45.14 (35.25–55.43)
Quintile 5 (Richest)	56	10.71 (4.79–22.23)	37	46.18 (30.76–62.38)	34	77.54 (59.06–89.21)	59	64.89 (50.65–76.89)	46	67.11 (49.21–81.12)	164	47.18 (35.79–58.86)	95	42.63 (31.06–55.07)	145	43.79 (32.67–55.57)	131	54.05 (41.44–66.17)
Education (4 groups)																		
No education	159	4.40 (2.04–9.25)	115	24.16 (17.12–32.95)	136	52.83 (43.66–61.82)	135	61.28 (51.26–70.42)	93	67.99 (57.02–77.29)	234	54.97 (45.68–63.92)	157	52.55 (43.18–61.75)	187	46.05 (37.14–55.21)	171	63.74 (56.08–70.76)
Primary	195	5.64 (2.95–10.52)	59	25.43 (15.52–38.77)	65	49.50 (36.95–62.11)	73	53.28 (41.68–64.53)	76	60.16 (47.98–71.20)	177	43.11 (32.65–54.22)	111	45.61 (35.70–55.88)	161	41.72 (32.18–51.91)	125	53.81 (43.07–64.20)
Secondary	–	–	116	40.95 (32.03–50.51)	104	56.87 (47.54–65.75)	1,690	51.48 (42.08–60.78)	131	59.29 (50.54–67.49)	375	41.43 (33.66–49.64)	262	55.33 (47.11–63.26)	434	40.84 (34.63–47.36)	446	46.21 (40.03–52.51)
Higher	–	–	–	–	–	–	–	–	–	–	–	–	32	49.02 (28.77–69.61)	48	53.32 (35.64–70.21)	84	67.01 (51.09–79.79)
Place of residence																		
Rural	280	4.29 (2.31–7.81)	217	25.92 (20.37–32.36)	212	45.24 (38.01–52.67)	235	51.49 (43.18–59.72)	181	57.49 (49.92–64.71)	474	43.30 (37.23–49.56)	320	53.89 (47.71–59.95)	489	45.84 (39.91–51.90)	459	55.97 (50.64–61.16)
Urban	99	10.10 (5.28–18.45)	75	47.76 (36.73–59.01)	95	71.69 (61.78–79.87)	148	61.41 (51.22–70.68)	126	70.45 (61.12–78.33)	344	48.91 (40.60–57.28)	241	50.15 (41.73–58.56)	341	38.70 (31.86–46.01)	367	49.52 (42.43–56.64)
Sex																		
Female	200	6.00 (3.42–10.32)	152	36.50 (29.15–44.54)	138	49.81 (40.96–58.67)	182	56.24 (47.29–64.81)	144	60.73 (52.11–68.73)	400	45.13 (38.00–52.47)	274	51.06 (44.16–57.91)	428	44.30 (38.43–50.33)	365	55.18 (48.56–61.62)
Male	179	5.59 (2.95–10.33)	139	26.07 (18.88–34.83)	170	56.35 (47.96–64.39)	202	54.51 (46.65–62.15)	163	64.65 (56.54–72.00)	418	46.16 (39.33–53.14)	288	53.44 (46.44–60.31)	403	41.43 (34.65–48.54)	462	51.47 (45.72–57.17)

LB, Lower Bound; UB, Upper Bound; Est., Estimate; N, Population.

### Inequality measures of estimates of factors associated with exclusive breastfeeding in Ghana, 1993–2022

The analysis reveals notable trends in inequality across various dimensions over time (Supplementary Table). Economic status (wealth quintile) exhibited a significant decrease in absolute disparities, with the absolute difference (D) dropping from 6.8% (95% CI: −2.5 to 16.1%) in 1993 to 5.2% (95% CI: −9.2% to −23.5%) in 2022, and the population attributable fraction (PAF) also decreased from 84.6% (95% CI: 83.4 to 85.8%) in 1993 to 1.8% (95% CI: 0.9 to 2.7%) in 2022, indicating a reduced role of wealth in inequality. Age inequalities remained minimal, with PAF values near zero, and the absolute differences (D) were relatively small, ranging from −1.5% in 1993 to 19.3% in 2017. Education showed slight increases in its contribution to inequality, with PAF values between 24 and 26%, reflecting a higher impact of education on disparities in later years. Residence (urban vs. rural) exhibited a decline in inequality, with the absolute difference (D) decreasing from 21.8% in 1998 to 2.4% in 2022, and the PAF reducing significantly from 74% to near zero. However, the subnational region showed the most persistent and significant inequality, with D values remaining high, reaching 50.6% (95% CI: 34.4 to 66.8%) in 2022 (Supplementary Table). This highlights that while economic and residential inequalities have decreased, regional disparities remain a significant source of inequality, emphasising the need for targeted policies addressing geographic and educational disparities.

## Discussion

This study examined trends and inequalities in exclusive breastfeeding in Ghana. At the national level, EBF prevalence among children aged 0–5 months in Ghana improved considerably, increasing from 5.8% in 1993 to 53.1% in 2022, with the highest rate of 62.8% recorded in 2008. The prevalence observed here is comparable to the 53.1% reported by Wepeba et al. (22) in northern Ghana. Nevertheless, it remains above the global average of 48% (1), reflecting significant national progress despite remaining gaps. These improvements may be attributed to the Baby-Friendly Hospital Initiative, national breastfeeding policies, and intensified community health education.

Maternal education emerged as a strong determinant of EBF practice. In 2022, women with higher education reported the highest prevalence (67.0%), aligning with findings from Nigeria, where women with secondary or tertiary education were more likely to practise EBF (23, 24). Although Ghanaian mothers with no formal education improved from 4.4% in 1993 to 63.7% in 2022, the persistence of disparities indicates that breastfeeding campaigns have not been uniformly effective. Strengthening equitable access to breastfeeding information and support, with particular attention to women who face literacy-related barriers to health communication, remains essential for achieving fair improvements across all population groups.

Economic status also influenced breastfeeding outcomes. In 2022, the poorest quintile achieved the highest prevalence (63.2%), overtaking the richest quintile (54.1%), which reverses earlier trends. Similar patterns have been reported in Ethiopia, where poorer households were more reliant on breastfeeding due to limited access to breastmilk substitutes (25). In contrast, wealthier and urban households are more likely to adopt formula feeding, often influenced by employment-related constraints and changing lifestyles (26). Within Ghana, consumption and labour-market statistics show that wealthier households tend to have greater access to commercial infant formula and higher female labour-force participation in formal sectors, where maternity protection remains limited to a minimum of 12 weeks (27, 28). These structural conditions shape inequalities in infant feeding and provide context for the socioeconomic gradients identified in the analysis. Further, these findings highlight the need for workplace lactation support, paid maternity leave, and broader family-friendly policies to sustain breastfeeding practices across all socioeconomic groups.

Age and sex-related differences were less pronounced. Adolescent mothers (15–19 years) reported lower EBF prevalence (49.9%) compared with older mothers (53.4%), although improvements from 7.1% in 1993 demonstrate progress. This pattern is consistent with evidence showing that young mothers often face barriers such as stigma, limited knowledge, and insufficient social support (29, 30). Infant sex differences were modest, with females (55.2%) slightly more likely to be exclusively breastfed than males (51.5%), similar to trends reported in India (31).

Although disparities existed across socio-demographic groups, these differences did not account for the apparent stalling of EBF over time. The magnitude of cross-sectional inequality was modest relative to the overall temporal increase, suggesting that population-level stagnation cannot be attributed solely to unequal progress among education, wealth, or regional groups. Furthermore, inequality did not follow a pattern of progressive widening. Instead, several dimensions, including economic status and residence, expanded during the early 2000s and subsequently narrowed in later years. This contraction indicates that disadvantaged groups did not consistently lag, and thus disparities were not the primary driver of the plateau observed after 2008. The levelling may reflect broader structural or programmatic factors that affected all population groups, rather than widening socio-demographic gaps.

### Implications for policy, research, and practice

The marked progress in EBF prevalence in Ghana between 1993 and 2022 demonstrates the impact of past initiatives, yet persistent regional and sociodemographic disparities necessitate renewed and inclusive policy approaches. The Ministry of Health and the Ghana Health Service should prioritise region-specific strategies, particularly targeting regions such as Western North, Central, and Eastern, where EBF rates remain below the national average. Policymakers must collaborate with the Ministry of Employment and Labour Relations to expand maternity protection policies, extend paid maternity leave, and enforce workplace breastfeeding-friendly practices, particularly in urban centres and among wealthier households where breastfeeding uptake lags. The Food and Drugs Authority and Parliament must further strengthen regulatory frameworks to limit the inappropriate marketing of breastmilk substitutes. Civil society organisations, including women’s advocacy groups, should be engaged to amplify breastfeeding-friendly social norms and community accountability, contributing to the achievement of SDG 3 on ensuring healthy lives and well-being for all.

The analysis reveals important gaps in knowledge requiring further scholarly attention. While national prevalence has improved, little is known about the contextual drivers of persistent regional and socio-economic inequalities. Future studies should explore determinants such as cultural norms, workplace practices, healthcare provider counselling, and the influence of commercial formula marketing. Longitudinal and mixed-method research is warranted to examine how changes in urbanisation, women’s education, and health system strengthening intersect to shape EBF practices. Additionally, there is a need for equity-focused evaluations of breastfeeding interventions, particularly in disadvantaged subnational regions. Disaggregated research by maternal age, education, and wealth status could provide nuanced insights into the intersectionality of inequalities. Generating such evidence will strengthen the evidence base for equity-sensitive programming in Ghana and other low- and middle-income countries.

Healthcare providers and frontline practitioners remain crucial in addressing disparities in EBF. Midwives, nurses, and community health workers should be trained to deliver context-sensitive counselling, with specific emphasis on adolescent mothers, urban households, and employed women who face unique barriers. The Community-based Health Planning and Services (CHPS) programme provides an important platform for embedding breastfeeding support into routine antenatal, postnatal, and child welfare services. Engagement of community leaders, religious institutions, and traditional authorities can enhance cultural acceptance and sustain behaviour change. Media houses and communication specialists should play a proactive role in designing campaigns that promote breastfeeding as both a child survival and maternal well-being strategy. Non-governmental organisations, including those focused on nutrition and maternal health, should continue to strengthen peer-support initiatives and provide practical assistance to mothers in marginalised communities.

### Strengths and limitations of the study

This study offers notable strengths. It draws on nationally representative Demographic and Health Survey data covering almost 30 years, providing a strong foundation for assessing long-term patterns in EBF. Disaggregation by maternal age, educational attainment, household wealth, child sex, residence, and region enables a detailed assessment of inequalities, while the use of advanced inequality metrics enhances understanding of population-level disparities. Subnational analysis further reveals geographic variation essential for targeted action. Several limitations must, however, be acknowledged. The cross-sectional design prevents causal interpretation, and reliance on maternal recall may introduce reporting bias. The absence of qualitative perspectives, including cultural norms, maternal knowledge, and structural barriers, limits contextual depth, and smaller sample sizes in certain regions may affect estimate precision. An additional methodological limitation relates to the use of indicators generated through the WHO HEAT. Evidence from Chabé-Ferret (32) shows that slight differences in definitions of EBF can lead to substantial inconsistencies across DHS and MICS, raising concerns about the robustness and comparability of standardised indicators. As this study relied entirely on HEAT-processed indicators, the results may reflect such definitional sensitivities. Nonetheless, the analysis offers valuable evidence to inform policy and future research.

## Conclusion

The findings reveal that exclusive breastfeeding in Ghana has risen markedly from 5.8% in 1993 to 53.1% in 2022, yet notable disparities remain across regions, education, wealth, and maternal age. Mothers with higher education and those aged 20–49 years consistently show higher uptake, though significant improvements have also been recorded among adolescents and women with no formal education. Socioeconomic trends have shifted, with the poorest quintile now surpassing the richest in breastfeeding rates, reflecting changing dynamics in infant feeding practices. Collectively, these patterns demonstrate remarkable progress in Ghana’s pursuit of Sustainable Development Goal 3 on maternal and child health, but also underscore the need for targeted, equity-focused interventions and regionally tailored strategies to address persisting gaps and ensure all infants benefit equally from exclusive breastfeeding.

## Data Availability

Publicly available datasets were analysed in this study. This data can be found at: https://www.who.int/data/inequality-monitor/assessment_toolkit.

## Glossary

ACI: Absolute Concentration Index
CHPS: Community-based Health Planning and Services
CI: Confidence Interval
D: Difference
DHS: Demographic and Health Surveys
EBF: Exclusive Breastfeeding
GDHS: Ghana Demographic and Health Survey
GHS: Ghana Health Service
GSS: Ghana Statistical Service
LB: Lower Bound
MICS: Multiple Indicator Cluster Surveys
MP: Multiprocessor (Stata/MP version)
PAF: Population Attributable Fraction
PAR: Population Attributable Risk
R: Ratio
SDG: Sustainable Development Goal
TX: Texas (location of StataCorp)
UB: Upper Bound
UNICEF: United Nations Children’s Fund
USA: United States of America
WHO: World Health Organization

## References

[1] UNICEF, WHO. Global breastfeeding scorecard 2023 rates of breastfeeding increase around the world through improved protection and support. Geneva: World Health Organization (2023):1–9.

[2] Wanjiru J Kerkula J Dushimirimana T Mieh S Curry T Mugisha J . Factors influencing exclusive breastfeeding in sub-Saharan Africa: analysis of demographic and health surveys. BMC Public Health. (2025) 25. doi: 10.1186/s12889-025-23045-z, 40375216 PMC12080165

[3] Mohammed S Oakley LL Marston M Glynn JR Calvert C. Time trends in the prevalence and determinants of age-appropriate breast feeding among children aged 0-23 months in Ghana: a pooled analysis of population-based surveys, 2003-2017. BMJ Open. (2022) 12:e059928. doi: 10.1136/bmjopen-2021-059928, 36008076 PMC9422843

[4] Ghana Statistical Service. Ghana multiple indicator cluster survey 2017/2018. (2019). Available online at: https://statsghana.gov.gh/gssmain/fileUpload/pressrelease/MICS SFR final_compressed.pdf (Accessed December 18, 2025).

[5] Ghana Statistical Services. Ghana demographic and health survey 2022: Key Indicattors report. Accra, Ghana, Rockville, Maryland, USA GSS ICF (2022):5–24.

[6] Mphasha MH Makwela MS Muleka N Maanaso B Phoku MM. Breastfeeding and complementary feeding practices among caregivers at Seshego zone 4 clinic in Limpopo Province, South Africa. Child (Basel, Switzerland). (2023) 10. doi: 10.3390/children10060986, 37371218 PMC10297182

[7] Hossain S Mihrshahi S. Exclusive breastfeeding and childhood morbidity: a narrative review. Int J Environ Res Public Health. (2022) 19:19. doi: 10.3390/ijerph192214804, 36429518 PMC9691199

[8] Masi AC Stewart CJ. Role of breastfeeding in disease prevention. Microb Biotechnol. (2024) 17:e14520. doi: 10.1111/1751-7915.14520, 38946112 PMC11214977

[9] Wallenborn J Levine G dos Carreira Santos A Grisi S Brentani A Fink G. Breastfeeding, physical growth, and cognitive development. Pediatrics. (2021) 147:8029. doi: 10.1542/peds.2020-008029 33888567

[10] Engelhart A Mason S Nwaozuru U Obiezu-Umeh C Carter V Shato T . Sustainability of breastfeeding interventions to reduce child mortality rates in low, middle-income countries: a systematic review of randomized controlled trials. Front Health Services. (2022) 2:889390. doi: 10.3389/frhs.2022.889390, 36925780 PMC10012727

[11] World Health Orgnization. Exclusive breastfeeding for optimal growth, development and health of infants. Geneva: World Health Organization (2023).

[12] Tahiru R Amoako M Apprey C. Exclusive breastfeeding: an exploratory thematic analysis of the perspectives of breastfeeding mothers and significant others in the tamale metropolis of northern Ghana. BMC Nutr. (2024) 10:161. doi: 10.1186/s40795-024-00973-4, 39696715 PMC11657459

[13] Amzat J Aminu K Matankari B Ismail A Almu B Kanmodi KK. Sociocultural context of exclusive breastfeeding in Africa: a narrative review. Heal Sci Reports. (2024) 7:e2115. doi: 10.1002/hsr2.2115, 38742092 PMC11089088

[14] Edney J Kovats S Filippi V Nakstad B. A systematic review of hot weather impacts on infant feeding practices in low-and middle-income countries. Front Pediatr. (2022) 10:348. doi: 10.3389/fped.2022.930348, 36147803 PMC9485728

[15] Mundagowa P Chadambuka E Chimberengwa P Mukora-Mutseyekwa F. Barriers and facilitators to exclusive breastfeeding practices in a disadvantaged community in southern Zimbabwe: a maternal perspective. World Nutr. (2021) 12:73–91. doi: 10.26596/wn.202112173-91

[16] Jiravisitkul P Thonginnetra S Kasemlawan N Suntharayuth T. Supporting factors and structural barriers in the continuity of breastfeeding in the hospital workplace. Int Breastfeed J. (2022) 17:87. doi: 10.1186/s13006-022-00533-1, 36536399 PMC9761035

[17] Balogun OO Dagvadorj A Anigo KM Ota E Sasaki S. Factors influencing breastfeeding exclusivity during the first 6 months of life in developing countries: a quantitative and qualitative systematic review. Matern Child Nutr. (2015) 11:433–51. doi: 10.1111/mcn.12180, 25857205 PMC6860250

[18] Ghana Statistical Service. Ghana 2021 population and housing census (2021) 44:1–128. doi: 10.1088/1751-8113/44/8/085201

[19] Ministry of Health. Holistic Assessment Report 2024. (2022). Available online at: https://www.moh.gov.gh/wp-content/uploads/2025/01/2023-Holistic-Assessment-Report.pdf (Accessed December 18, 2025).

[20] Ministry of Health. Ghana National Newborn Health Strategy and action plan 2014–2018. (2014). Available online at: https://www.healthynewbornnetwork.org/hnn-content/uploads/Ghana_Newborn_Flyer-FINAL.pdf (Accessed December 18, 2025).

[21] World Health Organization. Exclusive breastfeeding for optimal growth, development and health of infants. (2023). Available online at: https://www.who.int/tools/elena/interventions/exclusive-breastfeeding (Accessed December 18, 2025).

[22] Wepeba TW Dowou RK Akeriwe ML Wondong WP Abubakari A Aninanya GA. Exclusive breastfeeding determinants among healthcare professionals in northern Ghanaian hospitals: a cross-sectional study. J Health Popul Nutr. (2025) 44:226. doi: 10.1186/s41043-025-00977-1, 40611359 PMC12224623

[23] Sokan-Adeaga MA Sokan-Adeaga AA Sokan-Adeaga ED Osibogun A Edris H. Predictors of exclusive breastfeeding practice among nursing mothers attending a health care facility in a peri-urban setting in Lagos state. Nigeria Afr Health Sci. (2022) 22:545–59. doi: 10.4314/ahs.v22i2.63, 36407371 PMC9652619

[24] Esan DT Sokan-Adeaga AA Odesanya OE Akingbade O Awotunde TA Ramos CG. Breastfeeding patterns among mothers of different occupational groups in Ekiti state, southwestern Nigeria. J Public Health Res. (2025) 14:22799036251345536. doi: 10.1177/22799036251345537, 40491965 PMC12146593

[25] Woldeamanuel BT. Trends and factors associated to early initiation of breastfeeding, exclusive breastfeeding and duration of breastfeeding in Ethiopia: evidence from the Ethiopia demographic and health survey 2016. Int Breastfeed J. (2020) 15:3. doi: 10.1186/s13006-019-0248-3, 31924229 PMC6953467

[26] Mekonen EG. Bottle-feeding practice and its associated factors among mothers of children aged 0 to 23 months in sub-Saharan Africa: a multi-level analysis of demographic and health surveys (2015–2022). BMC Public Health. (2024) 24:1712. doi: 10.1186/s12889-024-19244-9, 38926817 PMC11209972

[27] Stumbitz B Kyei A Lewis S Lyon F. Maternity protection and workers with family responsibilities in the formal and informal economy of Ghana: practices, gaps and measures for improvement - International Labour Organization. ILO. (2017). Available online at: https://researchrepository.ilo.org/esploro/outputs/encyclopediaEntry/Maternity-protection-and-workers-with-family/995321029902676 (Accessed December 18, 2025).

[28] Dang H-AH Raju D Tanaka T Abanokova K. Poverty dynamics for Ghana during 2005/06–2016/17: an investigation using synthetic panels. Sci Afr. (2024) 25:e02282. doi: 10.1016/j.sciaf.2024.e02282

[29] Can V Bulduk M Can EK Ayşin N. Impact of social support and breastfeeding success on the self-efficacy levels of adolescent mothers during the postpartum period. Reprod Health. (2025) 22:19. doi: 10.1186/s12978-025-01960-z, 39905463 PMC11796182

[30] Coetzee B Tomlinson M Osawe S Abimiku A Kagee A. Barriers to and facilitators of adherence to exclusive breastfeeding practices among HIV infected and non-infected women in Jos, Nigeria. Matern Child Health J. (2017) 21:953–60. doi: 10.1007/s10995-016-2253-0, 28074312 PMC6086483

[31] Goyal K Purbiya P Lal S Kaur J Anthwal P Puliyel J. Correlation of infant gender with postpartum maternal and paternal depression and exclusive breastfeeding rates. Breastfeed Med. (2017) 12:279–82. doi: 10.1089/bfm.2017.0024, 28472601

[32] Chabé-Ferret B. Measuring breastfeeding prevalence using demographic and health surveys. BMC Public Health. (2024) 24:2366. doi: 10.1186/s12889-024-19821-y, 39217282 PMC11365256

