# Supplementary material for: Trends and inequalities in exclusive breastfeeding among 0–5 month olds in Ghana from 1993 to 2022
Source: Front Public Health. 2026 Jan 8;13:1702411. doi: 10.3389/fpubh.2025.1702411 (PMC12823812; doi:10.3389/fpubh.2025.1702411)
Supplement: Supplementary file 1 [file Table_1.docx]

**Table 2: Inequality measures of estimates of factors associated with exclusive breastfeeding in Ghana, 1993–2022**

|  |  | **1993** | | | **1998** | | | **2003** | | | **2006** | | | **2008** | | | **2011** | | | **2014** | | | **2017** | | | **2022** | | |
| --- | --- | --- | --- | --- | --- | --- | --- | --- | --- | --- | --- | --- | --- | --- | --- | --- | --- | --- | --- | --- | --- | --- | --- | --- | --- | --- | --- | --- |
| **Dimension** | **Measure** | **Est. (%)** | **CI-LB** | **CI-UB** | **Est. (%)** | **CI-LB** | **CI-UB** | **Est. (%)** | **CI-LB** | **CI-UB** | **Est. (%)** | **CI-LB** | **CI-UB** | **Est. (%)** | **CI-LB** | **CI-UB** | **Est. (%)** | **CI-LB** | **CI-UB** | **Est. (%)** | **CI-LB** | **CI-UB** | **Est. (%)** | **CI-LB** | **CI-UB** | **Est. (%)** | **CI-LB** | **CI-UB** |
| **Economic status (wealth quintile)** | D | 6.8 | -2.5 | 16.1 | 29.3 | 11.4 | 47.3 | 25.7 | 6.9 | 44.5 | 4.0 | -15.2 | 23.3 | 2.5 | -17.6 | 22.5 | -6.6 | -21.2 | 8.1 | -19.4 | -34.7 | -4.2 | -10.2 | -24.5 | 4.0 | -9.2 | -23.5 | 5.2 |
|  | PAF | 84.6 | 83.4 | 85.8 | 46.6 | 46.1 | 47.0 | 45.2 | 44.9 | 45.4 | 17.3 | 17.1 | 17.5 | 6.8 | 6.6 | 7.0 | 3.3 | 3.2 | 3.5 | 0.0 | -0.2 | 0.2 | 2.1 | 1.9 | 2.2 | 1.8 | 1.6 | 1.9 |
|  | PAR | 4.9 | -2.1 | 11.9 | 14.7 | -0.2 | 29.5 | 24.1 | 10.0 | 38.3 | 9.6 | -1.7 | 20.9 | 4.3 | -8.4 | 17.0 | 1.5 | -5.3 | 8.4 | 0.0 | -9.1 | 9.1 | 0.9 | -6.5 | 8.2 | 1.0 | -6.9 | 8.8 |
|  | R | 2.8 | 0.7 | 10.7 | 2.7 | 1.5 | 5.0 | 1.5 | 1.1 | 2.0 | 1.1 | 0.8 | 1.4 | 1.0 | 0.8 | 1.4 | 0.9 | 0.7 | 1.2 | 0.7 | 0.5 | 0.9 | 0.8 | 0.6 | 1.1 | 0.9 | 0.7 | 1.1 |
|  | ACI | 0.8 | -0.4 | 1.9 | 6.6 | 5.4 | 7.7 | 4.3 | -0.8 | 9.5 | 1.2 | -2.9 | 5.3 | 1.0 | -1.5 | 3.5 | -0.9 | -3.9 | 2.0 | -2.7 | -6.1 | 0.7 | -2.3 | -5.3 | 0.7 | -2.9 | -6.4 | 0.6 |
| **Population** | P | 379 |  |  | 291 |  |  | 308 |  |  | 383 |  |  | 308 |  |  | 818 |  |  | 561 |  |  | 830 |  |  | 826 |  |  |
| **Age (15–49)** | D | -1.5 | -9.7 | 6.7 | 14.7 | -1.1 | 30.6 | 11.4 | -6.3 | 29.2 | – | – | – | 8.7 | -11.7 | 29.1 | – | – | – | 19.3 | 1.9 | 36.7 | – | – | – | 3.5 | -11.5 | 18.6 |
|  | PAF | 0.0 | -0.2 | 0.2 | 4.6 | 4.5 | 4.6 | 2.1 | 2.0 | 2.1 | – | – | – | 1.3 | 1.3 | 1.3 | – | – | – | 2.8 | 2.8 | 2.9 | – | – | – | 0.5 | 0.5 | 0.6 |
|  | PAR | 0.0 | -0.9 | 0.9 | 1.4 | -0.1 | 3.0 | 1.1 | -0.8 | 3.0 | – | – | – | 0.8 | -1.0 | 2.6 | – | – | – | 1.5 | 0.3 | 2.7 | – | – | – | 0.3 | -0.7 | 1.3 |
|  | R | 0.8 | 0.2 | 2.6 | 1.8 | 0.8 | 4.2 | 1.3 | 0.9 | 1.9 | – | – | – | 1.2 | 0.8 | 1.7 | – | – | – | 1.6 | 1.0 | 2.5 | – | – | – | 1.1 | 0.8 | 1.4 |
|  | P | 379 |  |  | 291 |  |  | 308 |  |  | – |  |  | 308 |  |  | – |  |  | 561 |  |  | – |  |  | 823 |  |  |
| **Education (4 groups)** | D | – | – | – | – | – | – | -3.5 | -27.1 | 20.0 | – | – | – | – | – | – | – | – | – | -3.5 | -27.1 | 20.0 | 7.3 | -12.9 | 27.4 | 3.3 | -13.1 | 19.7 |
|  | PAF | – | – | – | – | – | – | 0.0 | -0.3 | 0.3 | – | – | – | – | – | – | – | – | – | 0.0 | -0.3 | 0.3 | 24.3 | 23.9 | 24.6 | 26.2 | 26.0 | 26.4 |
|  | PAR | – | – | – | – | – | – | 0.0 | -17.0 | 17.0 | – | – | – | – | – | – | – | – | – | 0.0 | -17.0 | 17.0 | 10.4 | -3.3 | 24.1 | 13.9 | 4.2 | 23.6 |
|  | R | – | – | – | – | – | – | 0.9 | 0.6 | 1.5 | – | – | – | – | – | – | – | – | – | 0.9 | 0.6 | 1.5 | 1.2 | 0.8 | 1.7 | 1.1 | 0.8 | 1.3 |
|  | ACI | – | – | – | – | – | – | 0.7 | -1.9 | 3.3 | – | – | – | – | – | – | – | – | – | 0.7 | -1.9 | 3.3 | -0.3 | -2.7 | 2.1 | -1.5 | -7.2 | 4.2 |
|  | p | 1112 |  |  | 872 |  |  | 920 |  |  | 1143 |  |  | 914 |  |  | 2421 |  |  | 1684 |  |  | 2491 |  |  | 2478 |  |  |
| **Residence (urban/rural)** | D | 5.8 | -0.9 | 12.6 | 21.8 | 9.3 | 34.4 | 26.5 | 14.9 | 38.0 | 9.9 | -2.9 | 22.7 | 13.0 | 1.6 | 24.3 | 5.6 | -4.8 | 16.0 | -3.7 | -14.2 | 6.7 | -7.1 | -16.4 | 2.2 | -6.4 | -15.3 | 2.4 |
|  | PAF | 74.0 | 73.2 | 74.8 | 51.6 | 51.3 | 51.9 | 34.2 | 34.1 | 34.4 | 11.0 | 10.9 | 11.1 | 12.2 | 12.1 | 12.3 | 7.1 | 7.0 | 7.2 | 0.0 | -0.1 | 0.1 | 0.0 | -0.1 | 0.1 | 0.0 | -0.1 | 0.1 |
|  | PAR | 4.3 | -0.2 | 8.8 | 16.3 | 6.5 | 26.0 | 18.3 | 9.9 | 26.6 | 6.1 | -0.2 | 12.3 | 7.6 | 1.2 | 14.1 | 3.2 | -0.8 | 7.3 | 0.0 | -4.8 | 4.8 | 0.0 | -4.0 | 4.0 | 0.0 | -3.8 | 3.8 |
|  | R | 2.4 | 1.0 | 5.6 | 1.8 | 1.3 | 2.5 | 1.6 | 1.3 | 1.9 | 1.2 | 0.9 | 1.5 | 1.2 | 1.0 | 1.5 | 1.1 | 0.9 | 1.4 | 0.9 | 0.8 | 1.1 | 0.8 | 0.7 | 1.1 | 0.9 | 0.7 | 1.1 |
|  | P | 379 |  |  | 291 |  |  | 308 |  |  | 383 |  |  | 308 |  |  | 818 |  |  | 561 |  |  | 830 |  |  | 823 |  |  |

**ACI – Absolute Concentration Index; D –Difference; LB – Lower Bound; PAF – Population Attributable Fraction; PAR – Population Attributable Risk; R – Ratio; UB – Upper Bound; P=Population**
